# A Handbook of the Theory and Practice of Medicine

**Published:** 1888-09

**Authors:** 


*A Handbook of the Theory and Practice of Medicine.* By FREDERICK T. ROBERTS, M.D., B.Sc., F.R.C.P. Seventh Edition. London: H. K. LEWIS. 1888.

When a book like Roberts's *Medicine* comes to us in its seventh edition, there is not much need to do more than chronicle the event. Of all handbooks to medicine there is perhaps none so generally useful as this. It is a book that leads the reader to think and observe for himself, rather than attempt to treat disease by consulting an index and using a ready-made prescription. This is the ground upon which we heartily recommend it.

